# Automated Segmentation of Trigeminal Nerve and Cerebrovasculature in MR-Angiography Images by Deep Learning

**DOI:** 10.3389/fnins.2021.744967

**Published:** 2021-12-10

**Authors:** Jinghui Lin, Lei Mou, Qifeng Yan, Shaodong Ma, Xingyu Yue, Shengjun Zhou, Zhiqing Lin, Jiong Zhang, Jiang Liu, Yitian Zhao

**Affiliations:** ^1^Department of Neurosurgery, Ningbo First Hospital, Ningbo, China; ^2^Cixi Institute of Biomedical Engineering, Ningbo Institute of Materials Technology and Engineering, Chinese Academy of Sciences, Ningbo, China; ^3^University of Chinese Academy of Sciences, Beijing, China; ^4^Department of Computer Science and Engineering, Southern University of Science and Technology, Shenzhen, China; ^5^The Affiliated People's Hospital of Ningbo University, Ningbo, China

**Keywords:** trigeminal nerve, cerebrovascular, segmentation, MRA, deep learning, coarse-to-fine

## Abstract

Trigeminal neuralgia caused by paroxysmal and severe pain in the distribution of the trigeminal nerve is a rare chronic pain disorder. It is generally accepted that compression of the trigeminal root entry zone by vascular structures is the major cause of primary trigeminal neuralgia, and vascular decompression is the prior choice in neurosurgical treatment. Therefore, accurate preoperative modeling/segmentation/visualization of trigeminal nerve and its surrounding cerebrovascular is important to surgical planning. In this paper, we propose an automated method to segment trigeminal nerve and its surrounding cerebrovascular in the root entry zone, and to further reconstruct and visual these anatomical structures in three-dimensional (3D) Magnetic Resonance Angiography (MRA). The proposed method contains a two-stage neural network. Firstly, a preliminary confidence map of different anatomical structures is produced by a coarse segmentation stage. Secondly, a refinement segmentation stage is proposed to refine and optimize the coarse segmentation map. To model the spatial and morphological relationship between trigeminal nerve and cerebrovascular structures, the proposed network detects the trigeminal nerve, cerebrovasculature, and brainstem simultaneously. The method has been evaluated on a dataset including 50 MRA volumes, and the experimental results show the state-of-the-art performance of the proposed method with an average Dice similarity coefficient, Hausdorff distance, and average surface distance error of 0.8645, 0.2414, and 0.4296 on multi-tissue segmentation, respectively.

## 1. Introduction

Trigeminal nerve is the fifth paired cranial nerves, and has the most complex nerve structures which contains three major branches: the ophthalmic nerve, the maxillary nerve, and the mandibular nerve. Trigeminal neuralgia (TN) is a typical chronic pain disorder caused by trigeminal nerve abnormalities, characterized by paroxysms of severe, lancinating pain in the distribution of the trigeminal nerveis, with an incidence of 4 per 100,000 population (Gronseth et al., 2008; Obermann, 2010). In general, TN involves the maxillary and mandibular nerve branches, and neurovascular compression is usually considered to be the main cause of primary trigeminal neuralgia. Common treatments for TN include percutaneous techniques, microvascular decompression, and Gamma Knife radiosurgery. In clinical practice, microvascular decompression (MVD) is considered to be the first choice and the gold standard of neurosurgical treatment (Cheng et al., 2014), where the vascular loop overlying the trigeminal nerve is displaced away from the root entry zone. This can be supported by a series of studies using Kaplan-Meier statistical analysis (Kaplan and Meier, 1958), that is, approximately 75% of patients treated with MVD can get a long-term cure (ie, no pain and no medication) (Bederson and Wilson, 1989; Barker et al., 1996; Broggi et al., 2000; Tronnier et al., 2001; Zakrzewska et al., 2005; Zhong et al., 2014).

The presence of neurovascular compression (NVC), the morphological and spatial relationships of vascular and nerve, the severity of the compression have significant impacts on the outcome of MVD (Li et al., 2004; Sindou et al., 2007; Yao et al., 2018). Accurate preoperative assessment of the characteristics of the NVC is not only critical to the surgical planning and the outcome of trigeminal neuralgia treatment, but also has the potential to shorten the operative time (Miller et al., 2008; Leal et al., 2010, 2011; Zeng et al., 2013; Xie et al., 2020). Visualization of the intracranial trigeminal nerve and cerebrovascularture will be beneficial in obtaining the characteristics of the NVC and morphological relationships. In consequence, accurate segmentation and 3D reconstruction of the trigeminal nerves and cerebrovasculature related to NVC are primary factors to improve the surgical planning and outcome of MVD surgery.

Several methods for visualization of neurovascularity within the root entry zone (REZ) have been proposed in recent years (Kumon et al., 1997; Yamakami et al., 2000; Fukuda et al., 2003; Anderson et al., 2006; Prieto et al., 2012). However, weaknesses such as low resolution and missing spatial structure of conventional two-dimensional structural MRI make these methods inadequately for constructing neurovascular spatial relationships in REZ. To overcome these deficiencies, Mikami et al. (2005) and Zhou et al. (2012) used 3D MRI that can provide high spatial resolution while delineating vessels and nerves (e.g., 3D fast imaging) to perform neurovascularity visualization. With the advantages of 3D MRI and the characteristics of different imaging modalities, some visualization methods based on multimodal image fusion have been applied and developed in clinical practice (Ohtani et al., 2016; Liu et al., 2020; Shi et al., 2021).

Although there are now many clinical efforts to diagnose trigeminal neuralgia based on 3D visualization, these methods are manually adjusted to show neurovascular structures in 3D space with the help of 3D visualization software such as 3D Slicer^1^ and BrainLab^2^. An obvious drawback is that these methods rely heavily on manual adjustment of various complex parameters to achieve better visualization. Therefore, the diagnosis of TN in this way is bound by the experience of the physician and results in a large human error, which is detrimental to the quantitative analysis of NVC. In addition, manual adjustment to visualize the REZ is time consuming, which severely slows down the development of a MVD surgical plan for TN patients and therefore is likely to miss the optimal state of treatment. Therefore, a precise, more effective, and fully automated NVC visualization and detection system with less impact from human error is needed.

In this paper, we propose a deep learning-based 3D volume segmentation framework to address the above limitations. The proposed method is an end-to-end segmentation network containing two stages: a coarse segmentation stage and a refinement stage. The former is used to obtain confidence maps of the trigeminal nerve, cerebral vasculature and brainstem with respect to the background, while the latter refines the boundaries of tissues/organs based on the confidence maps. Importantly, due to stronger robustness, higher accuracy and faster speed, the neural network-based segmentation algorithm effectively reduces human error in acquiring NVC morphological features and greatly speeds up REZ rendering, which also provides a more reliable basis for MVD surgery planning. Overall, this work makes the following contributions:

• To the best of our knowledge, this is the first fully automated work to reconstruct the trigeminal nerve, cerebrovasculature and brainstem segmentation in MRA imagery, so as to visualize the spatial information of NVC. Compared with existing semi-automatic methods based on auxiliary software, the proposed method receives higher accuracy and efficiency.

• We propose a novel end-to-end 3D segmentation model based on two stages—coarse segmentation stage and fine segmentation. The coarse segmentation stage produces a preliminary trigeminal nerve, cerebral vessels and brainstem segmentation results, and the second stage refines the preliminary results, to obtain more accurate overall segmentations.

## 2. Related Works

### 2.1. Semi-automatic 3D Visualization

Precise visualization of the anatomical features of NVC is essential to assist clinicians in diagnosis. Most of the existing studies are based on auxiliary tools to reconstruct neurovascular structure of TN patients. Wang et al. (2020) rendered the 3D volume based on the inverted h-T2WI image and manually captured the neurovascular anatomical features under man-machine interaction mode. Yamada et al. (2019) segmented the cerebrovasculature, trigeminal and brainstem by the commercial software Avizo (version 6.3.0, FEI, Portland, OR, USA), and then analyzed the hemodynamic characteristics of the diseased artery at the neurovascular compression site based on computational fluid dynamics (CFD). They demonstrated that CFD can be used as a useful clinical tool to determine the target of MVD under preoperative conditions. Danyluk et al. (2020) first converted DICOM images to NIFTI format, and then employed FMRIB's FSL (Smith et al., 2004) series toolbox to generate brain tissue (gray matter, white matter, cerebrospinal fluid) volume and an estimated intracranial volume (ICV), as well as determined volumes of bilateral hippocampus, amygdala, and thalamus. This work aims to explore the relationship between the volumes of the hippocampus and trigeminal nerve before surgery and TN. Manava et al. (2020) visualized various types of nerves including the trigeminal nerve through a series of direct volume rendering methods (Hastreiter et al., 2002; Naraghi et al., 2004, 2007) to analyze and compare different results of exact reproducible anatomical 3D-representations of the ventrolateral medulla (VLM) in patients with arterial hypertension. To address the feasibility and predictive value of multimodal image-based virtual reality in detecting and assessing features of neurovascular confliction, Yao et al. (2018) utilized the open source 3D Slicer to perform multimodal 3D images reconstruction to generate multimodal virtual reality (VR) images for detection of possible NVC in the cerebellopontine angle. To validate the accuracy of image-bsed pre-operative segmentation using the gold standard endoscopic and microscopic findings for localization and pre-opetative diagnosis of the offending vessel, Dolati et al. (2015) used BrainLab iPlanNet (BrainLab AG, Munich, Germany) to segment and reconstruct 20 3.0T MRI images with TN and hemifacial spasm. This study shows that image-based segmentation is a promising method that can be used to identify and locate offending vessels that cause hemifacial spasm and TN before surgery. Similarly, in Kin et al. (2009), computer graphics models were created with computer software and observed interactively for detection of offending vessels by rotation, enlargement, reduction, and retraction on a graphic workstation in patients with NVC syndrome. In general, many techniques such as Sánchez et al. (2010), Christiano et al. (2011), and Alsofy et al. (2021) for capturing NVC features in the clinic rely on manual operations based on semi-automated software.

### 2.2. 3D Medical Image Segmentation

Many computer vision methods have been developed and adopted to handle the 3D medical image segmentation tasks, including unsupervised methods and supervised methods. Early unsupervised methods (Zhang et al., 2016; Zhao et al., 2018; Ma et al., 2021) relied on manually pre-designed filters for image segmentation, including traditional methods based on statistical model analysis (Gao et al., 2011), phase field and likelihood models (Zhao et al., 2014), wavelet transform and Markov (Cao et al., 2016), and level sets (Hao et al., 2007). In a recent unsupervised medical image segmentation, Zhao et al. (2018) proposed a weighted symmetry filter for automatic 2D vessel enhancement and segmentation, and further extended it to the 3D case for vascular segmentation.

With the development of high-performance hardware devices, 3D medical image processing methods based on deep learning have been rapidly developed due to the superior feature extraction performance of neural networks. Çiçek et al. (2016) proposed a automated 3D tubular structural organ segmentation method based on 3D convolutional neural netowrks (CNNs). Zhang et al. (2019) proposed a novel method for 3D retinal optical coherence tomography angiography (OCTA) microvascular segmentation and surface reconstruction based on CNN. Sanchesa et al. (2019) proposed a Uception network based on U-Net-like architecture (Ronneberger et al., 2015) cooperate with Inception modules (Szegedy et al., 2016) to segment cerebrovasculature in MRA images. Milletari et al. (2016) further extended the U-Net-like architecture into a multi-layer densely connected convolutional network (named V-Net) to implement automatic segmentation of 3D medical volumes. Several cerebrovascular segmentation methods inspired by the human attention mechanism have also been developed. Zhang et al. (2020) proposed a convolutional neural network based on reverse edge attention mechanism (RE-Net) to perform 3D cerebrovascular segmentation and surface reconstruction. To capture detailed information of brain vessels at different resolutions, Ni et al. (2020) proposed a global channel attention model for brain vessel segmentation.

## 3. Methods

**Figure 1:**
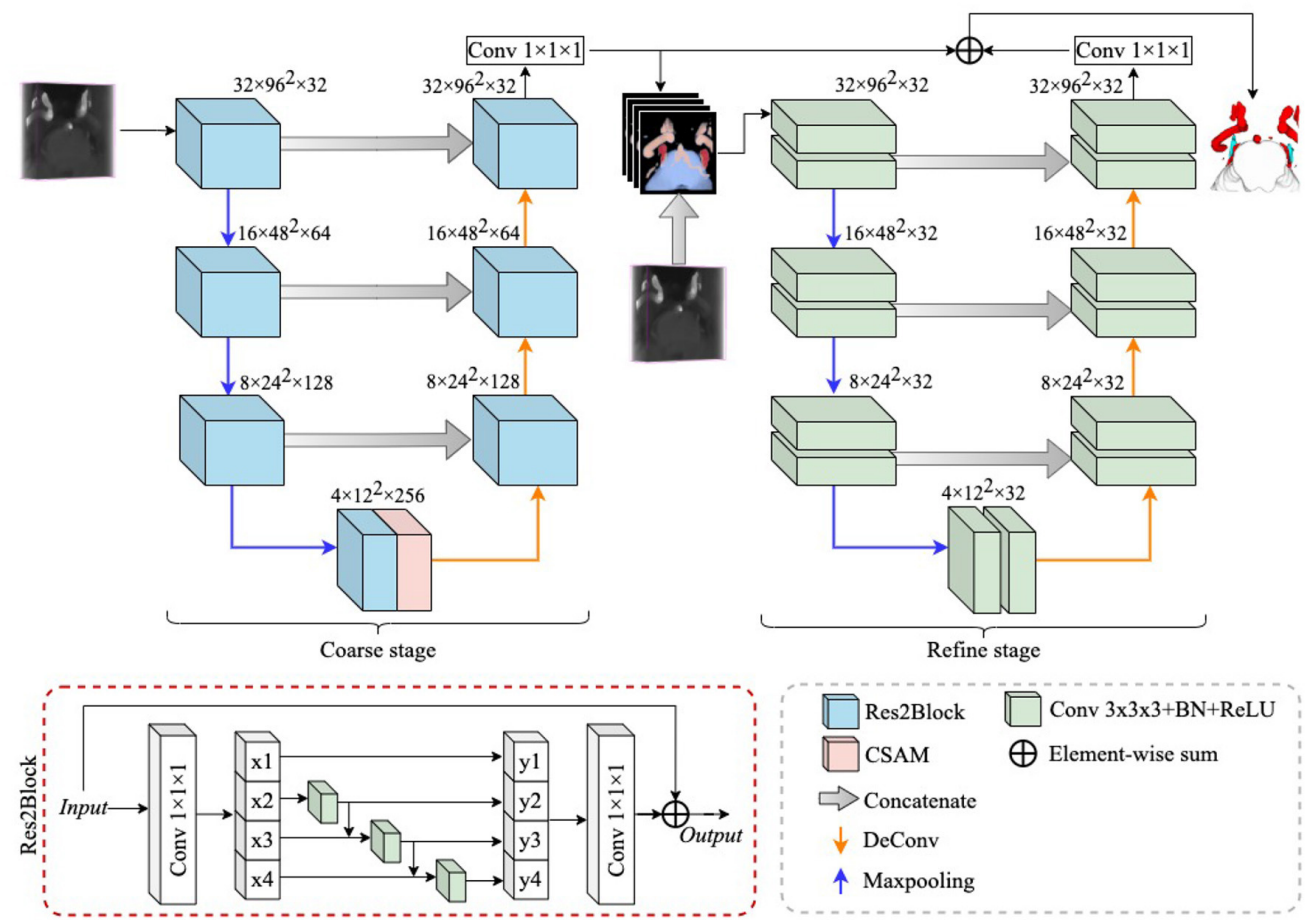
The overall structure diagram of the proposed method. The proposed method consists of a coarse stage and a refine stage, where the feature extraction module in the coarse stage is replaced by ResBlock to Res2Block, while the refine stage uses 3D UNet as a refiner to further improve the segmentation performance. The details of the structure of the CSAM can be found in CS^2^-Net (Mou et al., 2020).

In this section, we introduce a novel multiple tissue segmentation method based on 3D convolutional neural network, i.e., the segmentation of trigeminal nerve, cerebrovasculature and brainstem. Figure 1 illustrates the overall architecture of the proposed segmentation framework, which consists of two main components: 1) the coarse segmentation stage, and 2) the refine segmentation stage. The detailed parameter settings for each module are also listed in Figure 1.

### 3.1. Coarse Stage

Since the trigeminal nerve and cerebrovasculature are both tubular structures, we use the CS^2^Net (Mou et al., 2020) as the backbone in the coarse segmentation stage, and CS^2^Net was specifically designed for tubular structure segmentation. However, in order to achieve a more efficient feature extraction performance, we utilized the Res2Block (Gao et al., 2021) to replaced the residual feature extraction blocks (ResBlock) (He et al., 2016) in original CS^2^Net. On one hand, the Res2Block represents multi-scale features at a granular level, and increases the range of receptive fields for each network layers by constructing hierarchical residual-like connections within one single residual block. On the other hand, the input MRA volume contains small portion of trigeminal nerves, while the cerebrovascular are widely distributed. To this end, the powerful multi-scale feature extraction capability of Res2Block will benefit to the multi-scale feature learning of multi-scale tissues in MRA images.

Since this is a 3D volumetric tissue segmentation task, the original Res2Net cannot be imposed on the 3D case straightforwardly. Therefore, we replace the 2D convolutional layers in the original Res2Net with 3D convolutional layers. According to Gao et al. (2021), we divide the input features after the 1 × 1 × 1 convolutional layer equally into *s* subsets of feature maps along the channel dimension, and denote them as *x*_*i*_, where *i* ∈ {1, 2, ⋯ , *s*} and each *x*_*i*_ contains 1/*s* number of channels of the input feature channels. We apply a 3 × 3 × 3 convolution $K_{i}\left(\;\right)$, a batch normalization layer $B_{i}\left(\;\right)$ and a ReLU activation layer σ_*i*_() on each *x*_*i*_ accordingly, except *x*_1_. Thus, the operation on each subset *x*_*i*_ can be expressed as: $\sigma_{i}\left(B_{i}\left(K_{i}\left(x_{i}\right)\right)\right)$. Moreover, the feature subset *x*_*i*_ is added with the output of $\sigma_{i-1}\left(B_{i-1}\left(K_{i-1}\left(x_{i-1}\right)\right)\right)$, and then fed into $\sigma_{i}\left(B_{i}\left(K_{i}\left(x_{i}\right)\right)\right)$. Finally, we denote the output of each *x*_*i*_ as *y*_*i*_:


(1) $$y_{i}=\{\begin{array}{ll} x_{i} & i=1,\\ \sigma_{i}({ℬ}_{i}(K_{i}(x_{i}))) & i=2,\\ \sigma_{i}({ℬ}_{i}(K_{i}(x_{i}+y_{i-1}))) & 2<i⩽s. \end{array}$$

To further fuse the multi-scale information extracted by the Res2Block, we concatenate all output subsets, and pass them through a 1 × 1 × 1 convolution. The split and concatenation strategy can enforce convolutions to process features more effectively. In this work, we followed (Gao et al., 2021), and set *s* = 4.

### 3.2. Refine Stage

Due to the variable appearance and inconsistent size of multiple tissues, the boundaries of the trigeminal nerve, cerebrovasculature, and brainstem regions generated through the coarse segmentation stage are not clear, which greatly impair the credibility of NVC examinations that require clear visualization. Thus, it is crucial to provide clearer boundaries of different anatomical structures. Therefore, we add a module for refining the segmentation boundaries of different tissues after the coarse segmentation phase, which is named as the refine stage.

In detail, anatomical structures with unclear boundaries are fed into the refine stage to generate clearer anatomical structures. All voxels of anatomical structures with unclear boundaries are probabilized, which can be considered as a confidence map for different anatomical structures and is denoted as **P**, where **P** ∈ ℝ^4×*Z*×*H*×*W*^. 4 indicates that the confidence map has four channels, i.e., 4 classes, and *Z*, *H*, and *W* indicate volume thickness, height, and width, respectively. In addition to unclear boundaries, it is also possible that a small number of voxels of anatomical structures are not successfully identified, which can also impair the accuracy of NVC assessment. To address this limitation, we concatenate the raw volume (denoted as **I**) with **P** in the channel dimension, thus constructing a new input for the refine stage, denoted as **F**, i. e.,


(2) $$\begin{array}{l} \text{F}=\text{cat}\left(\text{I},\text{P}\right), \end{array}$$

where **F** ∈ ℝ^5×*Z*×*H*×*W*^. In this case, the new input **F** not only contains voxel confidence for different anatomical structures, but also complements the missing confidence with raw features. On the other hand, the confidence map imposed on the raw volume can be considered as a hard attention to the different anatomical structures, thus enhancing the segmentation performance of the framework.

In this work, we employ the 3D U-Net (Çiçek et al., 2016) as the backbone to extract hierarchical features, while the difference is that we set the channels in each layer of the 3D U-Net to 32, as shown in Figure 1. To reduce the loss of structural information, we summed **P** with the output features of the refine stage and then applied a 1 × 1 × 1 convolution to reduce the number of channels to 4. All extracted features first go through a coarse stage and then are fed into a refine stage, which allows the proposed framework to be trained and inferred in a end-to-end manner.

### 3.3. Loss Function

Supervised learning-based volumetric image segmentation requires a loss function during the learning process to measure the error between the predicted segmentation and the gold standard, so as to continuously optimize the segmentation performance. The loss function plays an important role in the feature learning process of neural networks. In this work, we segment the trigeminal nerve, cerebrovasculature, and brainstem regions from MRA images. However, the percentage of voxels of the trigeminal nerve in the MRA images is very small and is present in approximately 3 consecutive slices. One problem caused by the small percentage of trigeminal voxels is the imbalanced segmentation classes, which can lead the neural network to bias the learning of cerebrovasculature and brainstem features while ignoring the learning of trigeminal features during the training process. To address this limitation, we employ a multi-loss mixture function to measure the predicted segmentation of the network. In detail, we use the weighted cross-entropy loss ($L_{wce}$) and the Dice coefficient loss ($L_{dice}$) as the final loss function L for network training, i.e., defined as:


(3) $$\begin{array}{l} L=\alpha L_{wce}+\left(1-\alpha \right)L_{dice}, \end{array}$$

where α is a weight balance parameter between $L_{wce}$ and $L_{dice}$, which is empirically set as α = 0.5.

Weighted cross-entropy reduces the weights of other tissues and backgrounds and increases the weight of the trigeminal nerve in the central region to balance the effect of the trigeminal nerve with other tissues and backgrounds on the loss. In addition, the Dice loss function evolved from the Dice coefficient can reduce the sensitivity of the model to the imbalanced class and thus guide the network to obtain more semantic information about the trigeminal nerve. For our segmentation task, the weighted cross entropy loss function is defined as:


(4) $$\begin{array}{l} L_{wce}=-\overset{C}{\underset{i=1}{\sum }}\omega_{i}g_{i}\log p_{i}, \end{array}$$

where *C* indicates the number of classes, and where *g*_*i*_, *p*_*i*_, and ω_*i*_ are the truth label, normalized predicted probability, and class weight of *i*^*th*^ class, respectively. In this paper we set the class weights of background, brainstem, cerebrovasculature and trigeminal nerve as 1.0, 5.0, 20.0, and 300.0, respectively. Moreover, the $L_{dice}$ is defined as:


(5) $$\begin{array}{l} L_{dice}=1-\frac{1}{C}\overset{C}{\underset{i=1}{\sum }}\frac{2{\text{x}}_{i}^{2}{\text{y}}_{i}^{2}+\epsilon }{{\text{x}}_{i}^{2}+{\text{y}}_{i}^{2}+\epsilon }, \end{array}$$

where **x**_*i*_ and **y**_*i*_ represent the ground truth label and the predicted probability of *i*^*th*^ class, respectively, and where the ϵ is a Laplace smoothing factor used to avoid numerical instability problem, which is set as ϵ = 1.0 in this paper.

## 4. Material and Evaluation Metric

### 4.1. Dataset

**Figure 2:**
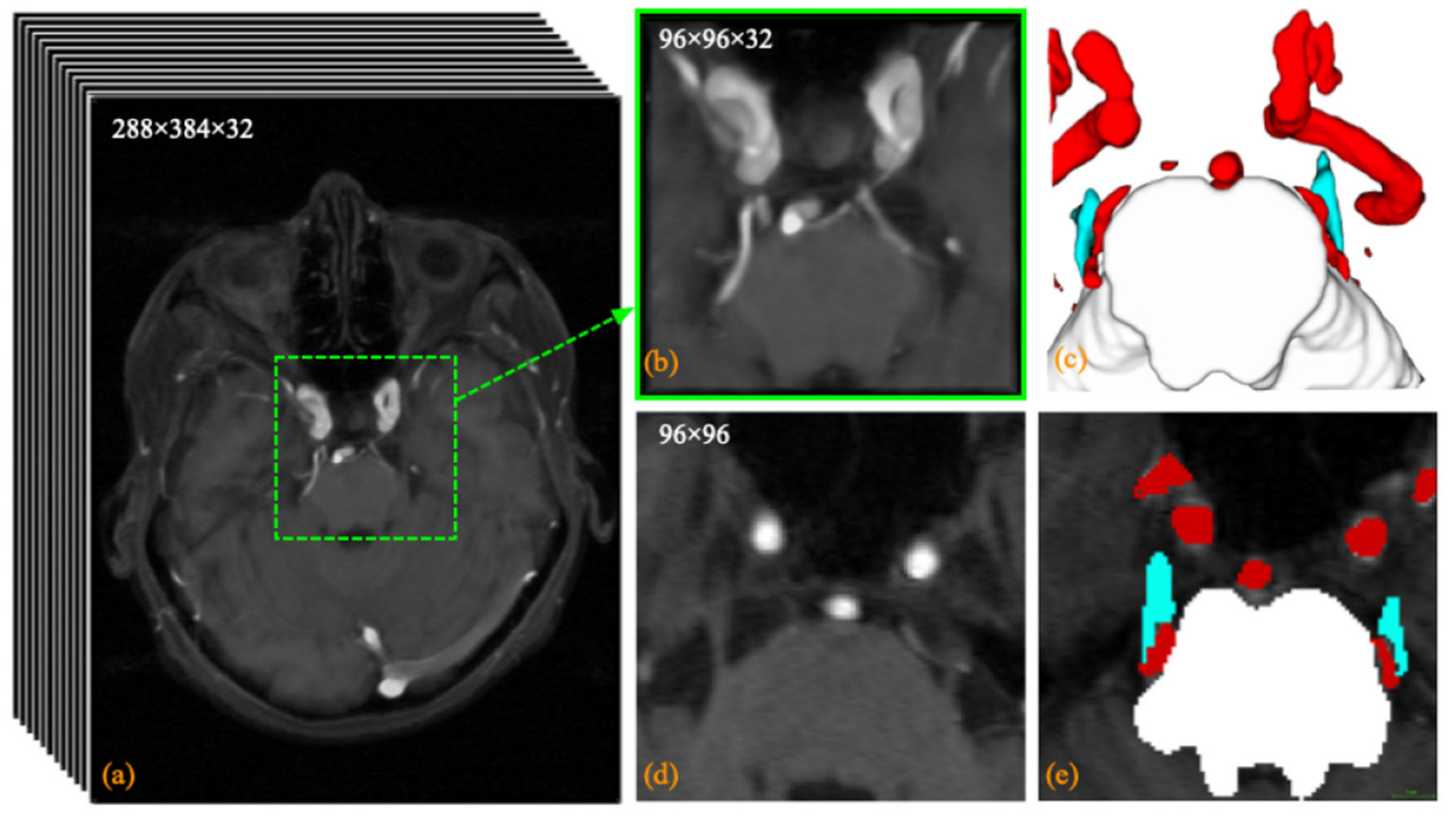
Visualization example of the trigeminal nerve and **(a)** the original MRA volume; **(b)** the enlarged view of the central area within the dashed green box; **(c)** the anatomical labeling of cerebrovascular, trigeminal nerve (blue) and brainstem; **(d)** the 14*th* slice of the MRA volume; and **(e)** the pixel labeling of cerebrovascular (red), trigeminal nerve (blue) and brainstem (white) in the 24*th* slice.

An in-house TRIgeminal NErve dataset (TRINE) acquired from local hospital, is used to train and validate the segmentation performance of cerebrovasculature and trigeminal nerves. This dataset aims to investigate the morphological relationship between cerebrovasculature and trigeminal nerves of patients with trigeminal neuralgia. All the MRA images were captured by a Siemens VIDA 3.0T machine by scanning trigeminal nerve area from 50 subjects, aged from 36 to 69 years. The voxel size is 0.5 × 0.5 × 1.0 *mm*^3^ and each volume consists of 288 × 384 × 32 voxels. To explore the morphological relationship between the trigeminal nerve and cerebrovasculature around the brainstem, the proposed method extracts three different tissues in acquired images, i.e., *brainstem, cerebrovasculature* and *trigeminal nerve*. Figure 2 shows a sample MRA volume from TRINE, and cerebrovascular (red), trigeminal nerves (blue) and brainstem (gray) have been annotated by expert.

### 4.2. Data Preprocessing and Manual Labeling

All raw data acquired from local hospital were stored in DICOM sequences. To facilitate input to the 3D convolutional network for learning, we exported the raw sequences into 3D metadata format. To circumvent the interference of tissue segmentation due to inconsistent spacing of the raw data for each patient, we resampled the metadata to a 1 × 1 × 1 spacing size. To reduce the GPU memory consumption for training and testing, we center-crop the original MRA volume at 96 × 96 × 32 size. This is because the REZ is located right in the center or not far from the center from the axial view. Therefore, we can directly center crop out the REZ without spending extra effort to detect the REZ. The manual annotation of cerebrovasculature and the trigeminal nerve of cropped volumes were labeled at pixel level by a senior neurosurgeon using software 3D Slicer^3^. Specifically, the neurosurgeon annotates the cerebrovascular by first obtaining a rough mask using the built-in thresholding module, then removing voxel clusters that are not cerebrovascular with the isolation module, and finally refining the boundaries to obtain an accurate cerebrovascular ground truth. For brainstem and trigeminal nerve labeling, the neurosurgeon generates the ground truth by manually outlining the boundaries and filling them in, respectively.

### 4.3. Evaluation Metrics

To quantitatively measure the performance of the proposed segmentation method, we use Dice similarity coefficient to measure the coverage between the segmented volume and the ground truth, which is defined as follows:


(6) $$\begin{array}{l} \text{DSC}=\frac{2|P\cap G|}{\left|P|+|G\right|}, \end{array}$$

where *P* and *G* represent the network predictions and corresponding ground truths, respectively. A higher DSC value indicates a superior segmentation performance, where higher than 0.70 generally indicates excellent agreement (Zijdenbos et al., 1994). Since DSC is sensitive to intra-volume overlay, and clear segmentation boundaries of cerebrovascular and trigeminal nerves are import for visualization of NVC. Therefore, the Hausdorff distance (HD) and the average surface distance (ASD) error are also employed as additional evaluation metrics, where HD is to measure the distance between the segmentation and the ground truth boundaries, while ASD is used to calculate the error between the segmentation and the ground truth surfaces. The HD metric is defined as follows:


(7) $$\begin{array}{l} \text{HD}\left(P,G\right)=\max\left(d_{PG},d_{GP}\right), \end{array}$$

where $d_{PG}=\underset{p\in P}{\max}\;\underset{g\in G}{\min}\;∥p,g∥$ is the Hausdorff distance between the boundary voxels in *P* and *G*. A smallier HD value indicates a more accurate segmentation. The evaluation metric ASD for quantifying surface distance error is defined as:


(8) $$\begin{array}{l} \text{ASD}=\frac{1}{2}\left(\frac{1}{n_{P}}\underset{p\in surf\left(P\right)}{\sum }d\left(p,G\right)+\frac{1}{n_{G}}\underset{g\in surf\left(G\right)}{\sum }d\left(g,P\right)\right), \end{array}$$

where *surf*(*P*) and *surf*(*G*) denote the surface of *P* and *G*, respectively. *n*_*P*_ and *n*_*G*_ are the total number of voxels in the *P* and *G* surfaces. *d*(*p, G*) denotes the closest Euclidean distance from voxel *p* on the surface of *P* to the surface of *G*. A smaller ASD value represents better segmentation performance.

## 5. Experimental Results

### 5.1. Experimental Setup

The implementation of the proposed method relies on the Python-based PyTorch^4^ (version = 1.5.0) framework with a dual NVIDIA GPU (Titan Xp). All training and testing procedures were run on a 32 GB RAM, Intel^Ⓡ^Xeon(R) Silver 4114 CPU @ 2.20GHz workstation. Adam serves as the optimizer for all comparative experiments. We adopt a poly learning strategy with an initial learning rate of 0.001 and a weight decay of 0.0005 to adjust the learning rate during training. The maximum number of iterations of the entire training procedure is set to 1,000, while the model with the best performance in 1,000 iterations is selected for evaluations. To evaluate the model fairly, we follow Mo and Zhang (2017) using a 5-fold cross-validation method for model testing, i.e., 40 volumes are used for training and the remaining 10 volumes serve for testing. In the training phase, we used a random flip with probability 0.5, and a random affine transformation with probability 0.5 for augmentation. Finally, the augmented volume is normalized to (0,1) after the generalization. In the test phase, we only apply normalization to the volumes.

### 5.2. Multi-Tissue Segmentation Results

To demonstrate the segmentation performance of the proposed method, we introduce several state-of-the-art 3D volume segmentation methods for comparison: 3D U-Net (Çiçek et al., 2016), V-Net (Milletari et al., 2016), Uception (Sanchesa et al., 2019), AnatomyNet (Zhu et al., 2019), and CS^2^-Net (Mou et al., 2020). 3D UNet and V-Net are the benchmark models in medical image segmentation, Uception and CS^2^-Net are state-of-the-art methods for cerebrovascular segmentation, and AnatomyNet is specialized with the segmentation of multiple organs. Therefore, the segmentation performance of the proposed method can be effectively demonstrated by comparing these typical methods. Table 1 demonstrates the comparison results of segmenting the brainstem (BS), cerebrovascular (CV), and trigeminal nerve (TN). In terms of DSC, the proposed method is inferior to CS^2^-Net in segmenting cerebrovascular. However, its segmentation of both brainstem and trigeminal nerve surpasses the state-of-the-art methods. In particular, for the segmentation of the trigeminal nerve, the DSC value of the proposed method exceeds that of AnatomyNet by 7.2%, which indicates that the proposed method is more advantageous in segmenting small tissue (trigeminal nerve). In addition, the proposed method remarkably improves the performance of trigeminal nerve segmentation in terms of Hausdorff distance as well as the average surface distance error, as evidenced by a 5.29 and 19.75% decrease in HD and ASD, respectively. Since the voxels of the trigeminal nerve account for a small portion of the cropped volume, yet the trigeminal nerve is a key component for visualizing NVC, it is important and challenging to accurately segment the trigeminal nerve. Thus, the proposed method focuses on extracting multi-scale features by introducing Res2Block. Finally, the proposed method achieved Dice similarity coefficients of 0.9344, 0.8508 and 0.8024 for brainstem, cerebrovascular and trigeminal nerve segmentation, respectively (all DSC values are higher than 0.7), which indicates that the segmented volumes of the proposed method are in excellent agreement with expert annotations. Furthermore, the smaller HD and ASD values demonstrate that the proposed method is able to segment the boundaries more clearly.

**Table 1:** Segmentation performance of comparison methods on MRA volumes.

**Methods**	**DSC**			**HD**			**ASD**			
	**BS**	**CV**	**TN**	**BS**	**CV**	**TN**	**BS**	**CV**	**TN**	**p**
3D U-Net	0.9096	0.7803	0.7213	0.2046	0.6451	0.3557	0.3119	0.9316	0.7904	0.001
VNet	0.8976	0.7888	0.5796	0.2790	0.5817	0.5136	0.3825	0.9127	1.1612	.001
Uception	0.8779	0.7821	0.6591	0.3051	0.6741	0.4648	0.4526	1.0258	0.9558	0.001
AnatomyNet	0.9305	0.8445	0.7304	0.1221	0.4879	0.2858	0.2225	0.7459	0.6831	0.001
CS^2^-Net	0.9321	**0.8603**	0.7117	**0.1096**	0.4096	0.3174	0.2158	0.6562	0.7171	0.029
**Proposed**	**0.9344**	0.8568	**0.8024**	0.1152	**0.3697**	**0.2329**	**0.2105**	**0.5927**	**0.4856**	

*BS, Brainstem; CV, Cerebrovascular; TN, Trigeminal nerve. Bolded values indicate the best performance*.

**Figure 3:**
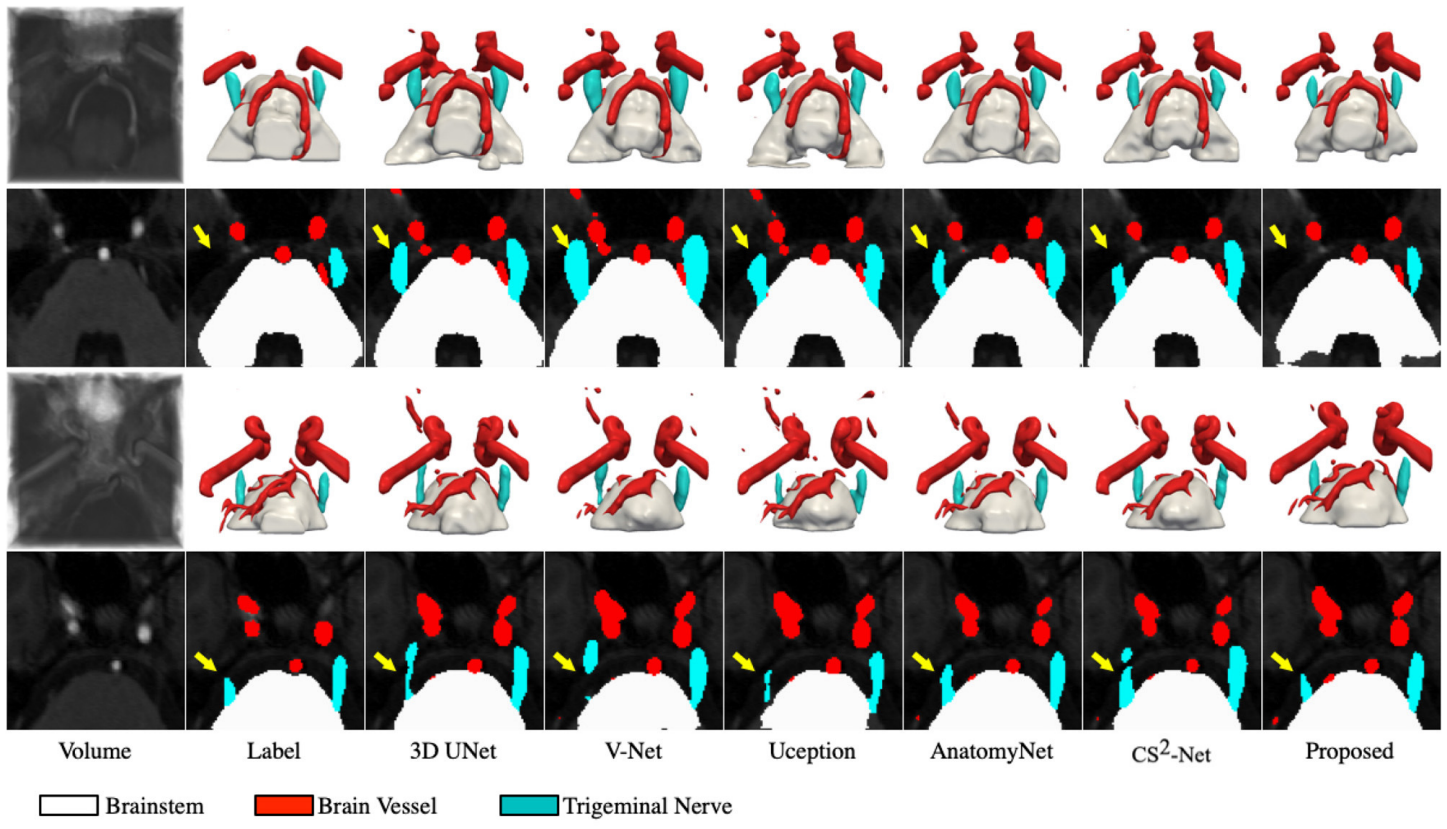
Visualization of the segmentation results. The first and third rows represents the 3D rendering of two segmentation volumes, and the second and fourth rows correspond to the 20 and 14*th* slices of the two segmentation volumes, respectively. From left to right in each column: the cropped MRA volumes and the corresponding intermediate slices, the ground truths, the segmentation results of 3D UNet, V-Net, Uception, AnatomyNet, CS^2^-Net and the proposed method.

To better demonstrate the superior performance of the proposed method to visualize NVC, we perform 3D rendering of the segmentation volumes of all comparison methods. Figure 3 illustrates the rendering results of two test samples, where the first and third rows are 3D renderings of the segmented volumes, and the second and fourth rows are the segmentation masks of the corresponding intermediate slices. By observing the 3D rendering in Figure 3, it can be seen that there are more isolated components in the segmented volumes of the comparison methods, especially in the cerebrovascular, while the proposed method produces smoother and more continuous tissues. Moreover, the superiority of the proposed method is further demonstrated by comparing the masks of the intermediate slices, as evidenced by the trigeminal nerve indicated by the yellow arrow in Figure 3. Specifically, 3D UNet, V-Net and Uception severely over-segmented the cerebrovascular and trigeminal nerve in the root entry zone, which is extremely detrimental to the subsequent diagnosis of NVC. Whether the trigeminal nerve and cerebrovascular vessels are under-segmented or over-segmented, their morphological structure is greatly impaired, which is not conducive to visualize NVC. If the trigeminal nerve in the root entry zone is not adequately segmented, the presence or absence of NVC cannot be fully observed. Therefore, the diagnosis of neurovascular compression may be confounded by over-segmented cerebrovascular and trigeminal nerve, meaning that it is possible that there is no real NVC, but only an artifact of over-segmentation.

### 5.3. Visualization of Neurovascular Compression

**Figure 4:**
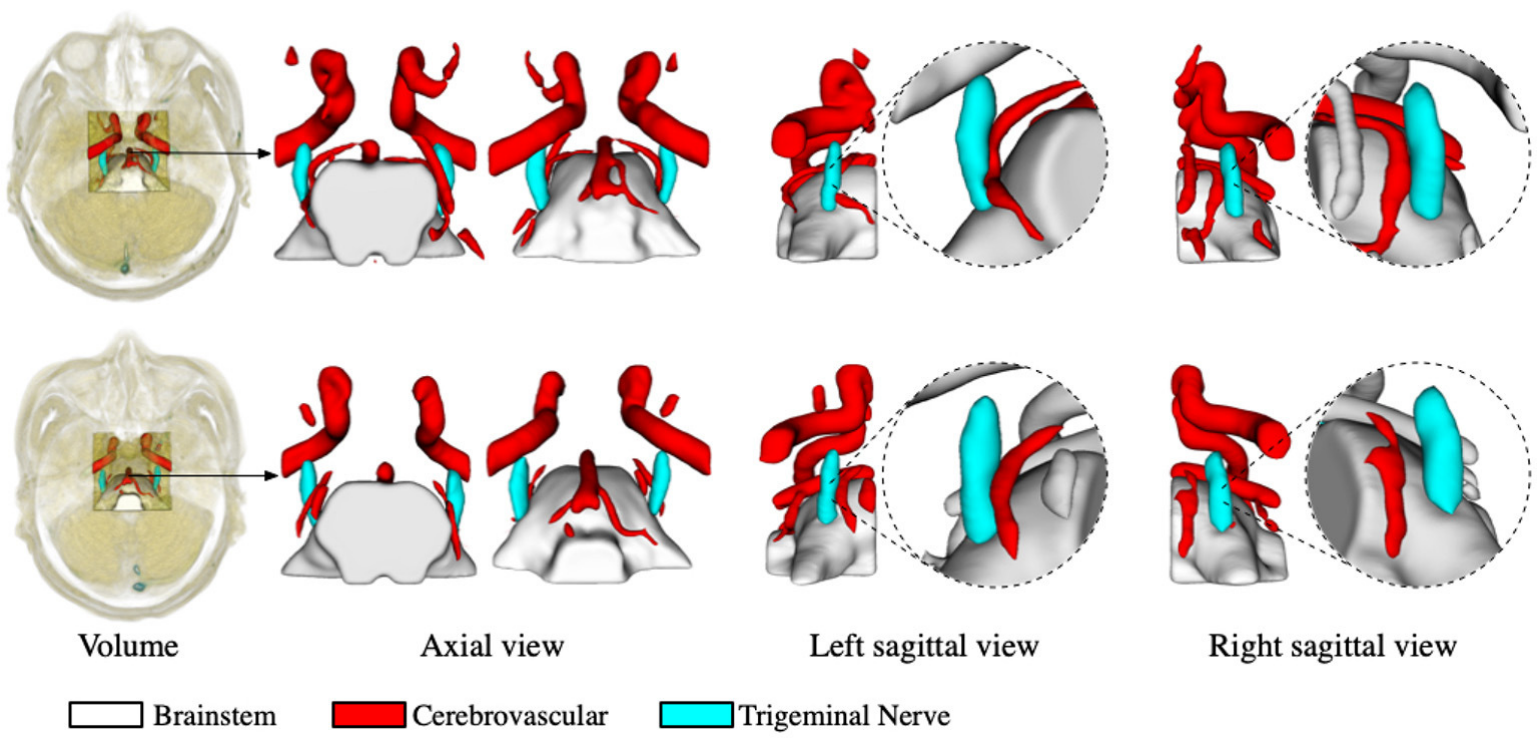
Virtual visualization of the root entry zone for TN patients. The two rows show patients with bilateral trigeminal neuralgia, and left-sided trigeminal neuralgia, respectively. The first column shows the location of the cropped volume in the original MRA, as well as the predicted anatomy of the cerebrovascular and trigeminal nerve. The second and third columns illustrate the rendering of the predicted anatomical structures in the axial view, while the last four columns indicate the rendering of the left and right perspectives in the sagittal plane and their enlarged renderings, respectively.

The segmentation of the trigeminal nerve and cerebrovasculature in the MRA volume ultimately serves the visualization of neurovascular compression. Therefore, in this section, we analyze the morphological structural spatial relationship between the local cerebrovasculature and trigeminal nerve in the root entry zone. Figure 4 shows two cases from the test set. The first and the second rows show patients with bilateral trigeminal neuralgia, and left-sided trigeminal neuralgia, respectively. The first column shows the location of the cropped volume in the original MRA volume of the brain and its anatomical structure of the cerebrovascular and trigeminal nerve labeled automatically by the proposed method. The second and third columns illustrate the 3D rendering of the anatomy in the axial view in the opposite perspective, while the last four columns indicate the 3D rendering of the anatomy on the left and right side of the sagittal view and its zoomed-in rendering in root entry zone, respectively. For the case in the first row, the enlarged rendering of the sagittal view shows that the left brain vessels and trigeminal nerve are extremely deformed due to compression, resulting in severe left-sided NVC, while the right-sided vessels and nerve are compressed but less severely deformed than the left, resulting in mild NVC, which is consistent with the clinical diagnosis. As for the second case, the rendering from its sagittal view visually shows that neurovascular compression exists on the left side while no compression is identified on the right side, which also coincides with the diagnosis of left-side trigeminal neuralgia.

## 6. Discussion

**Figure 5:**
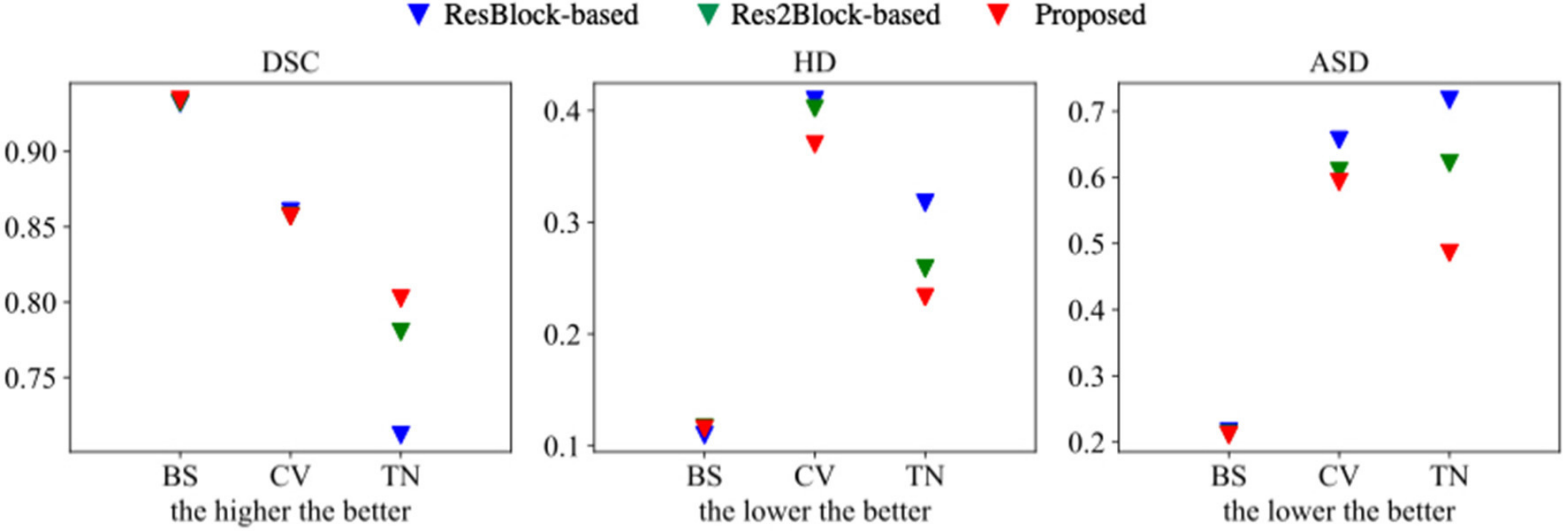
Segmentation performance of ResBlock-based CS^2^-Net, Res2Block-based CS^2^-Net and the proposed method.

The proposed multi-tissue segmentation method consists of a coarse segmentation stage and a fine segmentation stage, which utilizes an end-to-end training strategy to gradually refine the segmentation of trigeminal nerves and cerebrovasculature. The coarse stage employs a modified CS^2^-Net (Mou et al., 2020) as the backbone, i.e., we replace the original ResBlock with a Res2Block to improve the performance of the model for multi-scale feature extraction. And we introduce 3D U-Net (Çiçek et al., 2016) as the backbone network for refinement of cerebrovascular and trigeminal nerve boundaries in the fine stage. To verify the enhanced performance of the two-in-one segmentation framework for segmentation of the trigeminal nerve and cerebrovasculature, we conducted an ablation experiment to verify the performance of Res2Block and refinement backbone. Therefore, we train and test the ResBlock-based and Res2Block-based CS^2^-Net separately to obtain two sets of predictions. Then we train/test the Res2Block-based CS2Net embedded with a 3D UNet refiner. Figure 5 illustrates the comparison of the three ablation methods as mentioned above in terms of Dice similarity coefficient, Hausdorff distance, and average surface distance error. By observing the performance of the compared methods in terms of DSC, it is clear that the performance of ResBlock-based, Res2Block-based, and the proposed methods for segmenting the trigeminal nerve progressively improves, but unfortunately, the performance of segmenting the cerebrovascular slightly decreases. A plausible explanation is that the proposed method segmented the small cerebrovasculature that were under-labeled. Similar conclusions can be drawn from the performance of the HD and ASD aspects.

**Figure 6:**
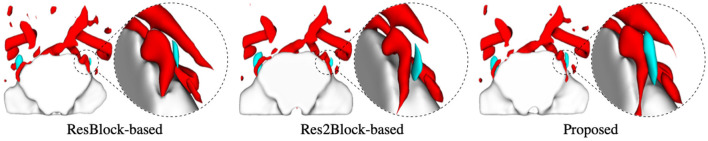
Local enlarge rendering of the trigeminal nerve in the right root entry zone.

To explore the importance of accurate segmentation of the trigeminal nerve for NVC visualization, we show 3D renderings of the volumes segmented by the ablation methods in Figure 6, respectively. As shown in Figure 6, the rendering circled by the dotted line indicates a local enlarged detail view of the trigeminal nerve. The local enlarged view demonstrates that the segmentation of the trigeminal nerve is increasingly accurate with the embedding of the Res2Block and 3D UNet refiner, which corresponds to the performance comparison shown in Figure 5. In other words, although the ResBlock-based and Res2Block-based methods can segment a portion of the trigeminal nerve, neither segmentation is complete enough. On the contrary, the proposed method can segment more trigeminal nerve voxels. Importantly, inadequate segmentation of the trigeminal nerve greatly increases the probabilities of under-diagnosis and misdiagnosis. For example, the patient represented in Figure 6 was diagnosed as right-sided trigeminal neuralgia by a clinical expert, yet both the ResBlock-based and Res2Block-based methods failed to diagnose the presence of NVC due to the inability to obtain the complete neurovascular relationship.

## 7. Conclusion and Future Works

Trigeminal neuralgia is a rare chronic pain disease with severe paroxysmal pain of the trigeminal nerve. Neurovascular compression (NVC) is generally considered to be the primary or most common cause of primary trigeminal neuralgia. Therefore, characterization of the NVC is one of the key steps in addressing trigeminal neuralgia. In order to better characterize the NVC of patients, we need a more accurate technique for segmenting the trigeminal nerve and its surrounding cerebrovasculature. In this paper, we propose a neural network-based method for trigeminal and cerebrovascular reconstruction, containing two stages: coarse segmentation and fine segmentation. The coarse segmentation stage is used to segment the original MRA volume, while the fine segmentation stage further refines the segmented map. The 3D reconstruction of the morphological structure of the trigeminal nerve and cerebral vessels greatly reduces the obstacle of obtaining their spatial relationship. The experimental results show that the proposed method can effectively observe and explore NVC. Our results indicate its potential of being applied as a powerful image analysis tool for computer-aided diagnosis and preoperative surgery simulation.

Although this paper highlights the potential of our proposed method for multi-class and especially small tissue segmentation, there are still several areas for improvement. The computational cost of 3D image segmentation is expensive, which leads to long model training and prediction times. Depending on the magnetic field strength, MRA imaging presents different modes, e.g., 1.5T MRA, 3.0T MRA. Since the segmentation models trained on fixed magnetic field strength MRA are difficult to generalize to other modal MRAs, cross-modal trigeminal nerve reconstruction is thus of significant research value. Currently, the proposed method detects only the neurovascular compression in the root entry zone. It is valuable to quantify more morphological features such as the degree of compression of the trigeminal nerve in the root entry zone.

## Data Availability Statement

The dataset analyzed for this study is not publicly available at this moment due to several legal regulations, further inquiries can be directed to the corresponding author.

## Ethics Statement

The studies involving human participants were reviewed and approved by Department of Neurosurgery, Ningbo First Hospital, Ningbo, China. The patients/participants provided their written informed consent to participate in this study.

## Author Contributions

JLin: conceptualization, data collection and annotation, formal analysis, literature research, methodology, validation, and writing—review. LM: software, methodology, validation, formal analysis, visualization, and writing—draft. JZ: supervision, writing—review, and editing. XY: data collection. SZ and ZL: resources and supervision. SM, QY, and JZ: supervision, writing—review, and editing. JLiu and YZ: conceptualization, resources, supervision, project administration, writing—review, and editing. All authors contributed to the article and approved the submitted version.

## Funding

This work was supported in part by the Zhejiang Provincial Natural Science Foundation of China (LZ19F010001), in part by the Youth Innovation Promotion Association CAS (2021298), in part by the Key Research and Development Program of Zhejiang Province (2020C03036), in part by the Ningbo 2025 S&T Megaprojects (2019B10033, 2019B10061, and 2020Z094), and Shenzhen Natural Science Fund (JCYJ20200109140820699 and the Stable Support Plan Program 20200925174052004).

## Conflict of Interest

The authors declare that the research was conducted in the absence of any commercial or financial relationships that could be construed as a potential conflict of interest.

## Publisher's Note

All claims expressed in this article are solely those of the authors and do not necessarily represent those of their affiliated organizations, or those of the publisher, the editors and the reviewers. Any product that may be evaluated in this article, or claim that may be made by its manufacturer, is not guaranteed or endorsed by the publisher.

## Footnotes

^1^ https://www.slicer.org/

^2^ https://www.brainlab.com/

^3^ https://www.slicer.org/

^4^ https://pytorch.org/
